# Assessing the role of participants in evolution of topic lifecycles on social networks

**DOI:** 10.1186/s40649-018-0054-x

**Published:** 2018-08-02

**Authors:** Kuntal Dey, Saroj Kaushik, Kritika Garg, Ritvik Shrivastava

**Affiliations:** 1 IBM Research, Vasant Kunj, New Delhi, 110070 India; 2 Department of Computer Science and Engineering, Indian Institute of Technology, Delhi, New Delhi, 110016 India; 3 Department of Information Technology, Ch. Brahm Prakash Govt. Engineering College, New Delhi, 110073 India; 4 Department of Information Technology, Netaji Subhas Institute of Technology, New Delhi, 110078 India

**Keywords:** Twitter topic lifecycle, Evolution of social topics, User influence in topic evolution, Semantic clusters of hashtags as Twitter topics

## Abstract

**Background:**

Topic lifecycle analysis on social networks aims to analyze and track how topics are born from user-generated content, and how they evolve. Twitter researchers have no agreed-upon definition of topics; topics on Twitter are typically derived in the form of (a) frequently used hashtags, or (b) keywords showing sudden trends of large occurrence in a short span of time (“bursty keywords”), or (c) concepts latent within the tweets that are grouped using variations of semantic clustering techniques.

**Methods:**

In the current paper, we jointly model the hashtags present and the semantic concepts embedded in the content, which in turn helps us identify hashtag groups that define a “topic”—a concept space—that are used by a large number of tweets.

**Results:**

We observe that different hashtags belonging to a given cluster are more prominent compared to the others, at different times. We further observe that the participation and influence levels of the different users play important roles in determining which hashtag would be more prominent than the others at given times. We thus observe topics to often morph from one to the other (via morphing of dominant hashtags representing the same semantic concept space), rather than becoming extinct outright, which is a novel insight about topic lifecycles. We further present novel observations about the role of users in determining the lifecycle of discussion topics on Twitter.

**Conclusions:**

We infer that topic lifecycles are governed by user interests, and not by user influence, which is a key observation made by our work.

## Background

Twitter has emerged as one of the most popular social network platforms with plethora of user-generated content, and one of the most explored ones by researchers. Works have attempted to characterize information diffusion, as well as to understand the dynamics of information diffusion over the social networks, along topics of discussion. Several models for information diffusion as well as methods to identify topics have been proposed in the literature.

A work by Ardon et al. [1] performed an early investigation around the lifecycle of topics on Twitter. As per their model, topics pass through a five-phase lifecycle, starting at the *pre-growth* (birth) phase, growing through the *growth* phase to reach the *peak* phase, and then eventually undergoing through the *decay* phase and dying down, around and after which a final *post* phase takes place. Other prior work in the literature has inspected Twitter hashtag lifecycle shape also. Using their K-spectral centroid (KSC) algorithm, Yang and Leskovec [2] characterize the temporal shape of usage of hashtags. However, the semantic and social angles are not explored by this work—it does not consider the semantics of the hashtags or the individual Twitter users (and their relationships) using those. It confines to examining the shape of the usage lifecycle of the hashtag. In an improvement over KSC, based on the hypothesis that semantically similar hashtags would temporal co-occur, the SAX algorithm was proposed by Stilo and Velardi [3]. While the SAX algorithm does consider the temporal overlap across hashtags (for deduplicating multiple hashtags), it neither considers the social aspect, nor does it look into the overlap across the semantic space across hashtags.

Clearly, the research of topic lifecycle on Twitter requires attaining further maturity. We recently performed a preliminary work to explore whether topic lifecycles can be better modeled and understood on Twitter, and reported our preliminary findings in Dey et al. [4]. However, further scope for research remains to better explore the social angle. A fundamental (social) question that requires exploration is *what is the impact of users in determining the lifecycle of topics?* In addition, there remains a scope to improve the definition of topics, while using a semantic-temporal definition framework. All of these would lead to significant novel insights, that have not been addressed in the earlier literature, thus motivating the current work.

Overall, this work explores evolution within topics, wherein one hashtag central to the topic is replaced by another, while users discuss a topic, on Twitter. Since the Twitter topic lifecycle literature simply treats each hashtag as a topic and assumes that a topic is past when a single hashtag is not used any more (or used very little), this study re-discovers the lifecycle of topics.

In this work, as part of topic detection of tweets, we perform semantic clustering of topics using word-embedding techniques. This approach is different from our earlier work [4], where the artifact used to perform hashtag clustering was Latent Dirichlet allocation (LDA) [5]. We define topics as a group of temporally proximal concepts that are semantically sufficiently similar, where the semantic similarity is derived using word-embedding similarity. This is different from the prior literature, where in absence of any universally agreed-upon definition, a topic is often defined as one of the following: (a) either simple hashtags (a hashtag is treated as a topic); (b) or bursty keywords, where a few keywords are used many times in a short span of time; or (c) sophisticated text-to-topic assignment techniques such as LDA.

Our renewed definition of topics enables us to analyze their lifecycle in a manner different from the rest of the literature, and we obtain novel insights. One of our key hypothesis is that, topics do not die; instead, they morph from one primary hashtag to another (which is usually a semantically and temporally related hashtag), and such evolution of the primary hashtag keeps happening for some time before the topic really dies down or morphs away into a different topic. A second key hypothesis is that, such evolution is enabled by the social mass participating in the topic, not by some highly influential single user. And further, we also hypothesize that the lifecycle of topics is correlated with the distribution of social connections. We explore real-life Twitter data, conducting experiments driven by the hypothesis above. Our experiments indicate that topic lifecycles are governed by user interests, and not by user influence, which is a first-of-its-kind insight in the space of topic lifecycle analysis on Twitter.

The rest of this paper is organized as follows. "Related work" section presents the literature in further detail. In "Central idea" section, we present the details of our approach. The experiments and observations are detailed in "Experiments" section. After a brief discussion in "Discussion" section, we conclude in "Conclusion" section.

## Related work

The literature for topic lifecycle analysis involves works around (a) identifying topics, (b) identifying topic characteristics for given periods of time, and (c) identifying the social association of the topic.

For detecting topics on Twitter, three techniques have been prominent in the literature. In the first, each hashtag is treated as a topic [6]. In the second, a set of (conceptually related) keywords that occur much more than the expected (average) occurrence within a short span of time are taken together to be treated as a topic [7, 8]. In the third, higher order semantic attributes of the tweet text are considered, and topics are assigned to the Twitter messages from the text, using advanced semantic-analysis mechanisms such as Latent Dirichlet allocation (LDA) [9–12]. While each of these methods have performed well in the context, they have been used in the literature, clearly, these have a shortcoming: these will not be able to identify contemporary and semantically related hashtags, such as, will not be able to identify that different tweets occurring around similar time periods, containing hashtags *#olympics*, *#olympicgames*, and *#theolympicgames*, all intend to address the same topic. Our work addresses this shortcoming, as one of its key contribution.

Ardon et al. [1] conduct an early work on understanding and analyzing Twitter topic lifecycle. They consider a hashtag as a topic, and model topic lifecycles as a five-phase phenomenon, starting at the *pre-growth* (birth) phase, growing through the *growth* phase to reach the *peak* phase, and then eventually undergoing through the *decay* phase and dying down, around and after which a final *post*-phase takes place. They augment their work by adding entities and places as hashtags, since a significant volume of tweets does not bear hashtags. Among other works, using their K-spectral centroid (KSC) algorithm, Yang and Leskovec [2] characterize the temporal shape of usage of hashtags. However, the work confines itself to the temporal shape of hashtag occurrence without considering temporal overlap of occurrence of two or more hashtags, and does not account for the semantic and social angles. The SAX algorithm, proposed by Stilo and Velardi [3], considers the temporal shape of the hashtag occurrence as well as temporal overlap of two or more hashtags. However, it neither considers the social aspect, nor does it look into the overlap across the semantic space across hashtags.

Twitter information diffusion is a well-studied area in general [13–15]. Discussion topics on Twitter, their social affinity, and geographical affinity have been addressed by the literature as well [16, 12]. User influence has also received significant research attention in the context of Twitter. Many works have attempted to determine user influence, and investigate the impact of such influence, such as [17–20]. An extensive survey of the literature, towards information diffusion and topic lifecycle analysis, has been conducted by Dey et al. [21].

We presented a preliminary version of our findings in an initial report [4]; however, the definition of topics used LDA and not the concept of word-embedding, as well as, the influence of users in the lifecycle of topics, has not been studied in the literature. This makes the current work novel, insightful, and valuable.

## Central idea

As described in "Background" section, the key contributions of this work lie in (a) modeling topic lifecycle, which captures the birth and growth of topics that are modeled as a cluster of semantically and temporally related hashtags, and the subsequent morphing of topics from being dominated by one hashtag to another related hashtag that in turn demonstrates continuity of the topic for a longer period than just an individual hashtag as well as (b) characterizing user influence in determining which hashtags that would dominate at given points in time. The technical approach of our work is described below.

### Identifying related hashtags

We identify conceptually (semantically) related hashtags using the “average word-embedding” of hashtags, as a simple average of embeddings of the words present in the group of tweets under consideration. We do this as a two-step process. First, for each hashtag present in the data, a document gets created. Second, for each hashtag, a word-embedding is created from its corresponding document. In principle, this approach is akin to Dey et al. [22].

Let $H = \{h_1, h_2, h_3, \ldots \}$ be the set of hashtags that appear across all the tweets under consideration. For each hashtag $h_i$, we retain all tweets $t_{h_i}$ the hashtag $h_i$, and consider these tweets together as a document:

1 $$\begin{aligned} D_{h_i} = \mathop {\bigcup }\{t_{h_i}\} - ({\forall h_i \in H})\{h_i\}. \end{aligned}$$

We create a “word-embedding” for each document created in the context of a hashtag. All hashtags occurring in document $D_{h_i}$, as well as mentions are eliminated. Let $W_{h_i} = \{w_{h_i}\}$ be the set of words appearing in $D_{h_i}$. Using a pre-trained embedding found on external resources (for our experiments, we use the popular GloVe [23]), we locate each word $w_{h_i}$ found in the input text (tweet). If found, we retain the word along with its embedding. Finally, the embedding $v_{h_i}$ for hashtag $h_i$, is computed as the average of all the word-embeddings present in the document:

2 $$\begin{aligned} v_{h_i} = \frac{\sum \nolimits _{w_{h_i} \in D_{h_i}}^{}(v_{w,h_i})}{|D_{h_i}|}. \end{aligned}$$

### Creating topic clusters

We perform semantic-temporal clustering process to create semantically related clusters of hashtags that occur temporally close enough. We perform *k*-means clustering based upon embedding distances of hashtag pairs, to segregate the hashtags into semantically related clusters. We apply temporal thresholds to create different groups for the different hashtags that do not get used (temporally) around similar times.

#### Semantic relationship building

As indicated earlier, a *k*-means clustering approach is used for identifying hashtag clusters. The distance between a given pair of hashtags is defined as the embedding distance of the two vectors representing the two respective hashtags. Embedding distance can be computed as any well-known distance function of a pair of vectors; in our experiments, we use cosine similarity of the embeddings of the hashtag pair, such that the higher the cosine similarity between the pair of hashtags, the lower is the distance. Formally, the cosine similarity between a *d*-dimensional vector pair *u* and *v* is computed as

3 $$\begin{aligned} {\mathrm{{cosine\_similarity} = cos}}(\theta ) = \frac{\sum \nolimits _{i=1}^d{u_iv_i}}{\sum \nolimits _{i=1}^d {u_i^2}\sum \nolimits _{i=1}^d {v_i^2}}.\end{aligned}$$

Using the results of vector pair similarity as distance values, we execute *k*-means clustering on the hashtags, thereby producing semantically related hashtag clusters, $T_s$.

#### Temporal relationship building

The clusters generated by the semantic relationship building process ensures creating clusters of hashtags that have been used in tweets with semantic similarity. However, it is important to ensure that the hashtags are also clustered such that they co-occur temporally, within permissible time periods, so that the clusters only contain hashtags that can lead to a potential continuity of discussion topics (if those topics are at all discussed by the social network members). We apply Allen’s temporal functions [24] on the semantically related clusters, to retain the temporally coherent clusters. In Allen’s jargon, the *overlap* relationship denotes instances, where there exists some (non-zero) co-occurrence of a given pair of events, the *meets* relationship captures, where one event starts as soon as another one stops, and the *disjoint* relationship represents when one event starts after another one is over with no common point of occurrence in time.

The time series for the individual hashtags, as well as the semantic clusters of the hashtags derived earlier, are developed. We compute whether a hashtag, as well as some hashtag(s) from its cluster, was used (or not) in a given time slot. A semantically related hashtag pair $h_i$ and $h_j$ are said to be temporally related if the pair satisfies the *overlaps* or *meets* relationships, or if they are *disjoint* but not by more than a threshold time period (we set this value to 2 days for experiments). Furthermore, the pair is also said to be related if at least another hashtag $h_k$ exists, such that $h_k$ is temporally related to $h_i$, and the hashtag pair $h_k$ and $h_j$ satisfies at least one of the temporal relationships (*overlaps*, or *meets*, or, within a threshold, *disjoint*). This makes the temporal relationship recurrent in nature. Furthermore, note that a pair of hashtags $h_i$ and $h_j$ are said to be unrelated if $\not \exists h_k$, such that $h_i$ is temporally related to $h_k$, and the hashtag pair $h_k$ and $h_j$ share an *overlaps*, *meets*, or, within a permissible threshold, *disjoint* relationship. Formally, the temporal relationship is given as

4 $$\begin{aligned} h_i \odot h_j \implies \Big ((\exists h_k) h_i \odot h_k\Big ) \cap (h_k \circledcirc h_j). \end{aligned}$$

Here, $\odot$ and $\circledcirc$ capture the *temporally related* and *overlaps* relationships, respectively. A semantic cluster $T_s$ with *m* different temporal relationships will be correspondingly split into *m* clusters, namely, $T_{s,t_1}$, $T_{s,t_2}$, ..., $T_{s,t_m}$.

The topics are defined as semantic-temporally related hashtag clusters, and are computationally finalized as clusters of hashtags that are related semantically. As an intuitive example, we expect the hashtags to be clustered together as long at they occur closely enough in time (if these are detected to occur in semantically similar tweets), such as we expect a cluster to contain hashtags such as {#clinton, #hilaryclinton, #clintonelection2016}, and a different cluster to contain a different group of hashtags altogether, such as {#bobdylan, #johndenver, #harrybelafonte}.

### Analyzing topic lifecycles

Prior literature [1] indicates events (topics) to be distributed over five-phase lifecycles. It starts at the *pre-growth* (birth) phase, where the topic is born in (or, enters into) the network, grows through the *growth* phase to reach the *peak* phase, where “early majority” discuss it, undergo the *decay* phase (where ‘late majority” discuss it) and die down, around and after which a final *post*-phase takes place, where “laggards” discuss it. The literature treats given hashtags, as well as hashtags assigned via natural language processing (NLP) techniques, as topics.

Our work, though, only uses the tweets with given hashtags, especially given the relatively low state-of-the-art accuracy in the space of hashtag prediction on Twitter [22], since predicting hashtags is not the main objective of our work. The choice of data set, thus, guarantees the presence of a minimum of one hashtag in each tweet under consideration. The topics we create, using the techniques described above, will tend to comprise of multiple hashtags. We explore along two angles.

#### Topic morphing detection over dominant hashtags

Let a given topic cluster $z_k$ comprise of a subset of hashtags $H_k = \{_kh_1, _kh_2, \ldots , _kh_m\}$ that have been used at least once, in a given time slot. Let the counting function *c* count the number of uses of a hashtag within a given time slot in cluster $z_k$. The dominant hashtag—the hashtag used with the highest frequency within the cluster $z_k$ at the time slot—will be given as

5 $$\begin{aligned} _kh_x = \forall (i) \left(\text{max }(c(_kh_i))\right). \end{aligned}$$

Effectively, at a given slot of time, the dominant hashtag is the “most representative hashtag” of a given topic cluster. It is interesting to observe that the individual hashtags (dominant as well other hashtags) tend to follow the observations made by Ardon et al. [1] as far as their lifecycles are concerned. However, although the lifecycles of individual hashtags are shorter, one hashtag is often replaced by another belonging to the same hashtag cluster, thereby entailing the topic to have a different dominant hashtag. This phenomenon effectively ensures that the topic continues its lifecycle via the morphing process over a set of semantically and temporally related hashtags, although the presence of the individual hashtags that characterized the topic earlier might have significantly reduced, or even have ceased to be further used.

#### Intensity detection for topics

If for a given topic cluster $z_k$, the subset of hashtags $H_k = \{_kh_1, _kh_2, \ldots , _kh_m\}$ happen to have been used at least once in a given time slot, and if *c* is the counting function for the number of uses of each hashtag $_kh_i$, then, the intensity of the topic is computed as

6 $$\begin{aligned} _kh_x = \sum \limits _{i=1}^{m}\left(c(_kh_i)\right). \end{aligned}$$

Thus, we define the intensity of a given topic as the cumulative use of all participant hashtags belonging to the cluster, in the time slot. In our experiments, we conduct a thorough study of topic licecycles (hashtag clusters), which analyzes the individual hashtags as well as the cluster of hashtags that define the topic as a whole, and further examine one with contrast to the other. We observe the morphing and intensity of the topics (clusters of hashtags) manifested by the individual hashtags, thus studying topics as a whole all over their lifecycles, as well as the usage of the individual hashtags at the different stages of their lifecycles, namely early, mid and late stages.

### User timeline creation for topic participation

We further create user timelines for their participation in topics, and analyze their roles in determining the topic lifecycles. We determine the participation of each user in a topic, by identifying whether they post tweets that contain at least one hashtag that is a part of the topic’s hashtag cluster. For each time slot, we find the participation of each user in a given topic. If user *u* makes a tweet having hashtag ${h_i}$ belonging to hashtag cluster $z_k$ in time slot $t_s$, then the topic participation in $z_k$ of *u* is up-counted. The process is repeated for all users, across all hashtags. If a hashtag contains multiple tweets belonging to the same topic at the same time slot, then the user’s participation in the topic at that time slot is multiply counted. This completes the creation of user timeline for topic participation.

### Assessing user roles in topic lifecycles

Using the user timeline created for their participation in the topics at each of the time slots, we proceed to assess the roles they play in the topic lifecycles. We determine the influence of the individual users in the overall graph, using their social friendship connections (for our experiments, we use Twitter followership). We use the well-known Page Rank [25] to measure user influence. Page Rank of user $u_i$, given a set of *n* users $U = \{u_1, u_2, \dots , u_n\}$, is given as

7 $$\begin{aligned} {\text{ PR }}(u_i) = \frac{1-d}{n} + d \sum \limits _{u_j \in \Gamma _{in}(u_i)}\frac{{\text{ PR }}(u_j)}{\Gamma _{\text{ out }}(u_j)}.\end{aligned}$$

Here, $\Gamma _\text {in}$ and $\Gamma _\text {out}$ represent the number of incoming and outgoing links, respectively, and *d* is a damping factor that avoids transforming the entire Page Rank of a user to its neighbors. We further perform hashtag usage profiling of users for each time slot, by calculating the total number of users that (a) use only the dominating hashtag, (b) use both dominating and non-dominating hashtags, and (c) use only non-dominating hashtags. We correlate these hashtag usage profiles of the users with their Page Ranks, to obtain an overall understanding of the role and influence of users towards the dominance (and non-dominance) of given hashtags at given slots of time. We perform this study over the entire lifecycle of given hashtag clusters (topics) as well as individual hashtags, to obtain a complete understanding of the role of users in the topic lifecycles.

### Graph characterization

We perform two types of characterizations of the social network graph with respect to the topic lifecycles. In the first, we examine the graph-level metrics of the topic graphs, such as connected components (strong and weak), degree distribution and graph diameter. In the second, we examine the user-level characteristics with respect to the topic graphs, such as page rank and user participation distributions for the topics. These are well-known graph metrics, and we assume prior familiarity of the reader to these metrics.

## Experiments

We perform our experiments using the models developed in "Central idea" section. The details of our experiments and observations are provided in this section.

### Data set description

We use the Twitter data set by Yang et al. [2] at Stanford^1^, which contains 20–30% of all the tweets posted on Twitter within the collection period. Without loss of generality, we randomly select the first two sets of tweets within these groups of collections: (a) those made in the last 20 days in June 2009 and (b) all the tweets made in July 2009. The social network graph connections are made available by in Kwak et al. [14].^2^ To avoid using over-popular hashtags and under-used ones, and to ensure that the experiments account for the “often-enough” used hashtags, in each data set, we retain only all the tweets containing at least one hashtag that has been used anywhere between 40 and 1000 times. The users making these tweets are retained, and we use the social network connections among the retained users to form their subgraph. Table 1 shows our data sets.

**Table 1:** Description of available data

Total num. of tweets	Tweets retained	Users retained	Hashtags retained	Edges retained	Avg. num. tweets/user	Connections per user
18,572,084	196,250	69,198	1843	1,380,569	2.84	19.95
46,130,269	854,036	205,394	7316	5,882,913	4.16	28.64

The tweets are from June and July 2009, respectively

### Experimental setup

We perform the hashtag clustering using the semantic and temporal techniques, as disclosed in "Central idea" section. We vary the range of k in the *k*-means clustering process, since there is no well-agreed upon way to determine a universally good value of k in the literature. For the June data, we detect, respectively, 50, 100, 150, ..., 450 and 500 topics (10 different granularities, at step sizes of 50). For July, we detect 200, 400, 600, ..., 1800 and 2000 topics (10 different granularities, at step sizes of 200), respectively. In other words, to maintain consistency of observations, we experiment over 10 different clustering granularities that range approximately from slightly higher than 2.5% of the data size, leading to slightly less than 40 hashtags per cluster on an average (thus, a relatively loose definition of a topic), to slightly higher than 25% of the data size, leading to slightly less than 4 hashtags per cluster on an average (thus, a relatively tight definition of a topic). This is followed by timeline creation for individual hashtags, for topics (hashtag clusters) as a whole and for users. We set the duration of each time slot as 1 h. This sets up the platform for performing the main experiments.

### Topic lifecycle characteristics: topic intensities and dominant hashtags

Table 2 provides a set of intuitive examples of the topic clusters formed, chosen randomly from the available pool. These examples will provide the reader with an intuitive view of hashtags forming the clusters.

**Table 2:** Examples of randomly chosen clusters with randomly chosen *k*-values (*k* of *k*-means clustering)

k-value	Cluster content
	*Example clusters from June 2009 data*
500	#trends, #trendy, #trend, #fashion
400	#icon, #Revolution, #freedom, #election, #revolution, #world
300	#Cricket, #PakCricket, #T20, #pakcricket, #cricket, #t20
200	#recipes, #food, #gfree, #cooking, #nom, #primal, #foodie, #vegan, #recipe
100	#wedding, #vintage, #etsy, #flowers, #makeup, #jewelry, #gardening, #zazzle, #artfire, #twittographers, #beauty, #giveaway, #handmade, #Etsy, #shoes, #etsytwitter
	*Example clusters from July 2009 data*
2000	#Swineflu, #pandemic
1600	#Weather, #weather, #Denmark, #lightning, #Copenhagen
1200	#mashup, #remix, #soul, #HipHop, #mv, #chill
800	#Ford, #automotive, #Cars, #car, #Remart, #ford, #hybrid, #used, #Car, #Autoparts
400	#demilovatolive, #DLovatoOnUstream, #DisneyRecords, #JBsouthamerica, #DemiLovatoLive, #DemiLiveWebcast

**Fig. 1:**
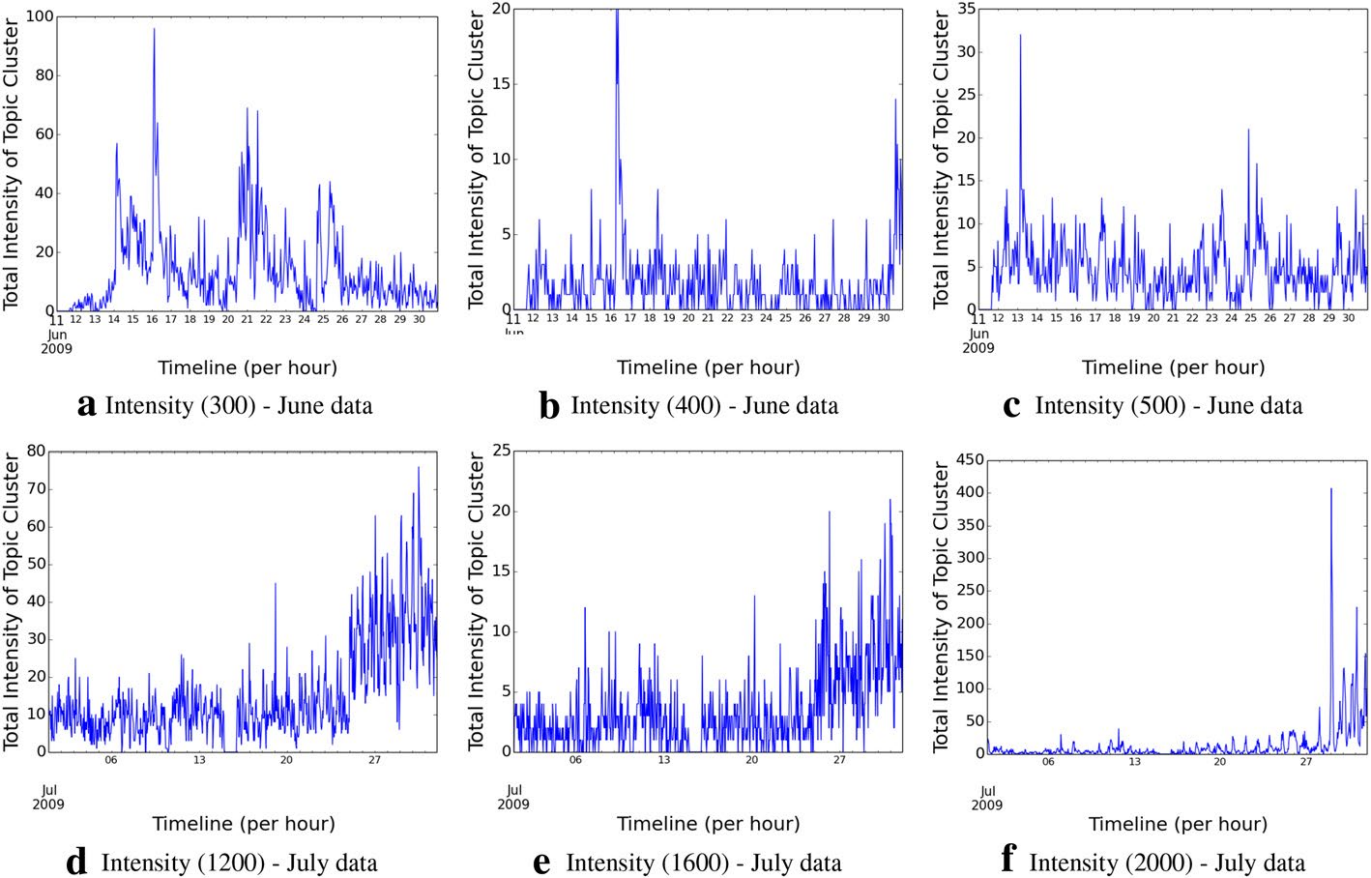
Topic intensity over time across the two data sets

The lifecycle of a topic is characterized by its different intensities and different dominant hashtags, over its period of existence. We explore the topic intensity characteristics over the time period, in Fig. 1, using some randomly chosen examples. Clearly, for both the data sets, we observe that after topics get born, they go through a cycle of peaks and troughs, as the intensity of using individual hashtags attain their crest, and subsequently reduce, while other semantically related hashtags pick up within the permissible time boundaries.

**Fig. 2:**
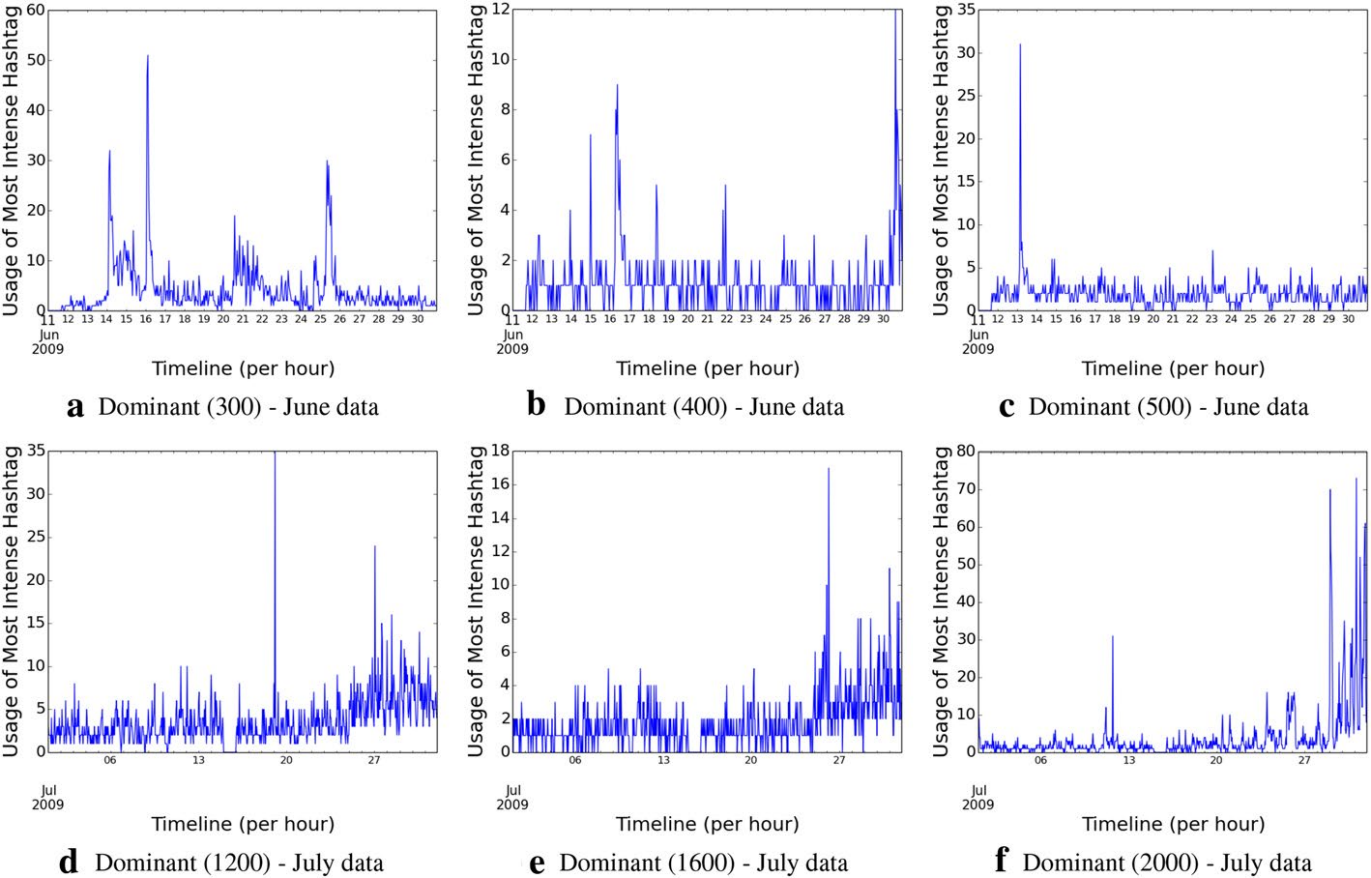
Temporal evolution of dominant hashtags across the two data sets

We further explore the hashtag dominance patterns over time, across different hashtags. We illustrate the patterns in Fig. 2 using the same examples that were randomly chosen to show the intensities (so that the reader can correlate the patterns across the two metrics); however, our inspection reveals the presence of similar characteristics across both the data sets. The patterns support our hypothesis, that, as one hashtag falls in terms of usage over time, other semantically related hashtags often become dominant within permissible time boundaries, and that determines the longevity and characteristics of the topic, defined by the cluster of hashtags, as a whole.

### Qualitative study of an example topic

**Fig. 3:**
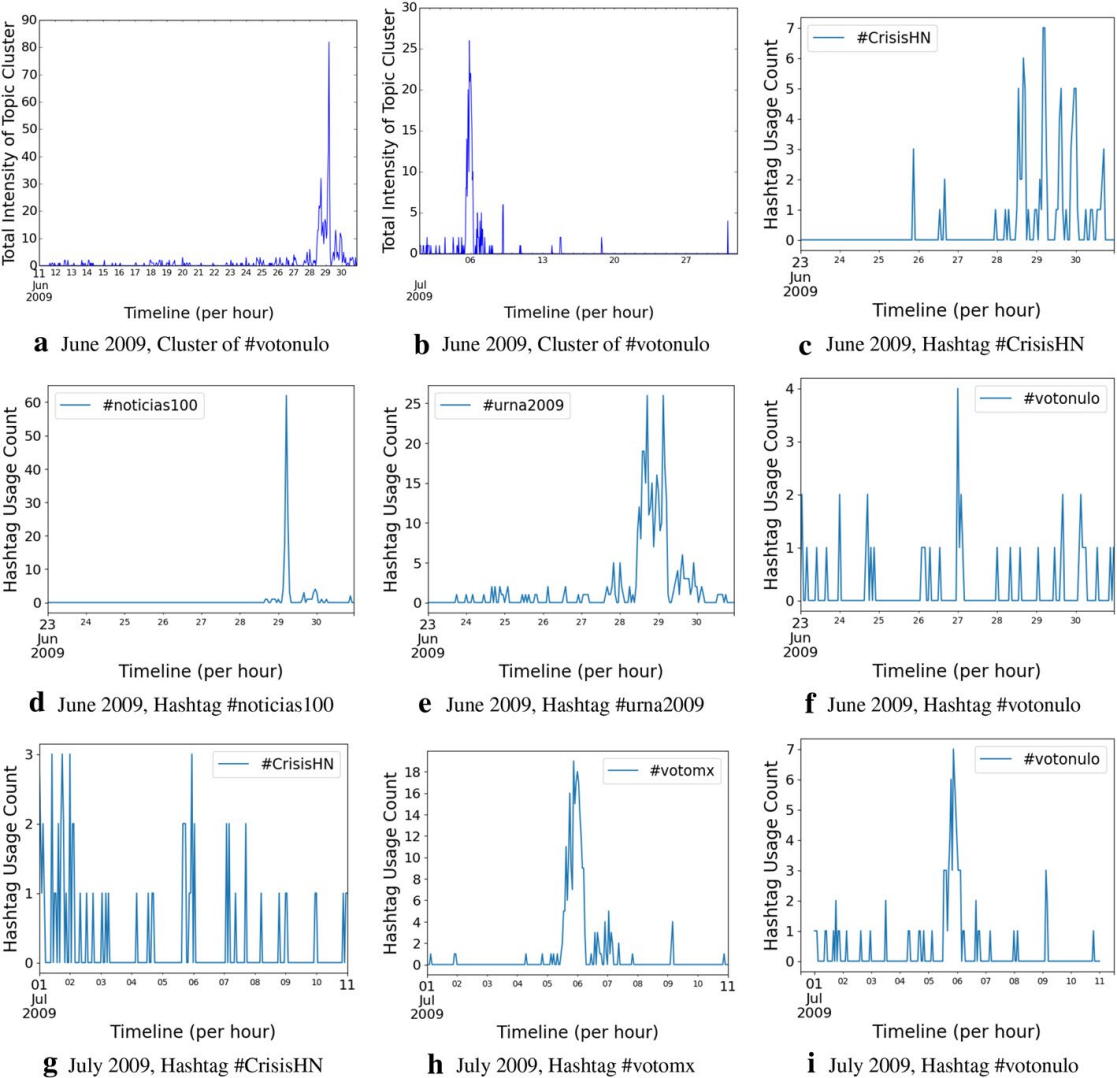
Qualitative analysis of lifecycle of the hashtag **#**votonulo, using the clusters and hashtags it associates with in June and July 2009

To provide a deeper level of intuition to the reader over a longer period, we pick a random hashtag from the hashtags that span across the 2 months, June and July, and show that while individual hashtags do not last long (which, until date, has been considered to be the lifespan of a hashtag-based topic in the literature), the hashtag cluster lasts much longer, and morphs from one hashtag to another. This phenomenon has been captured in Fig. 3.

As an example of a hashtag that spans across 2 months of our data set, we randomly select the hashtag **#**votonulo. This hashtag is born around 23rd June 2009 and dies around 11th July 2009. It connects the hashtags #CrisisHN, #noticias100 and #urna2009 in June 2009, and #votomx and #CrisisHN in July 2009, thus semantically connecting the evolving concepts. Note that, the dominating hashtag in June 2009 for this cluster has primarily been #noticias100 and #urna2009 and in July 2009 has been #votomx, while #votonulo and #CrisisHN have been non-dominant. We further observe in the data that the hashtags #noticias100 and #urna2009 do not even exist in July 2009, having died by then, and the hashtag #votomx does not exist in June, getting born in early July 2009. This example thus provides the reader a demonstration of how the hashtags undergo birth and death, but semantic topics continue for longer than the lifespan of the topic.

### User participation characteristics

**Fig. 4:**
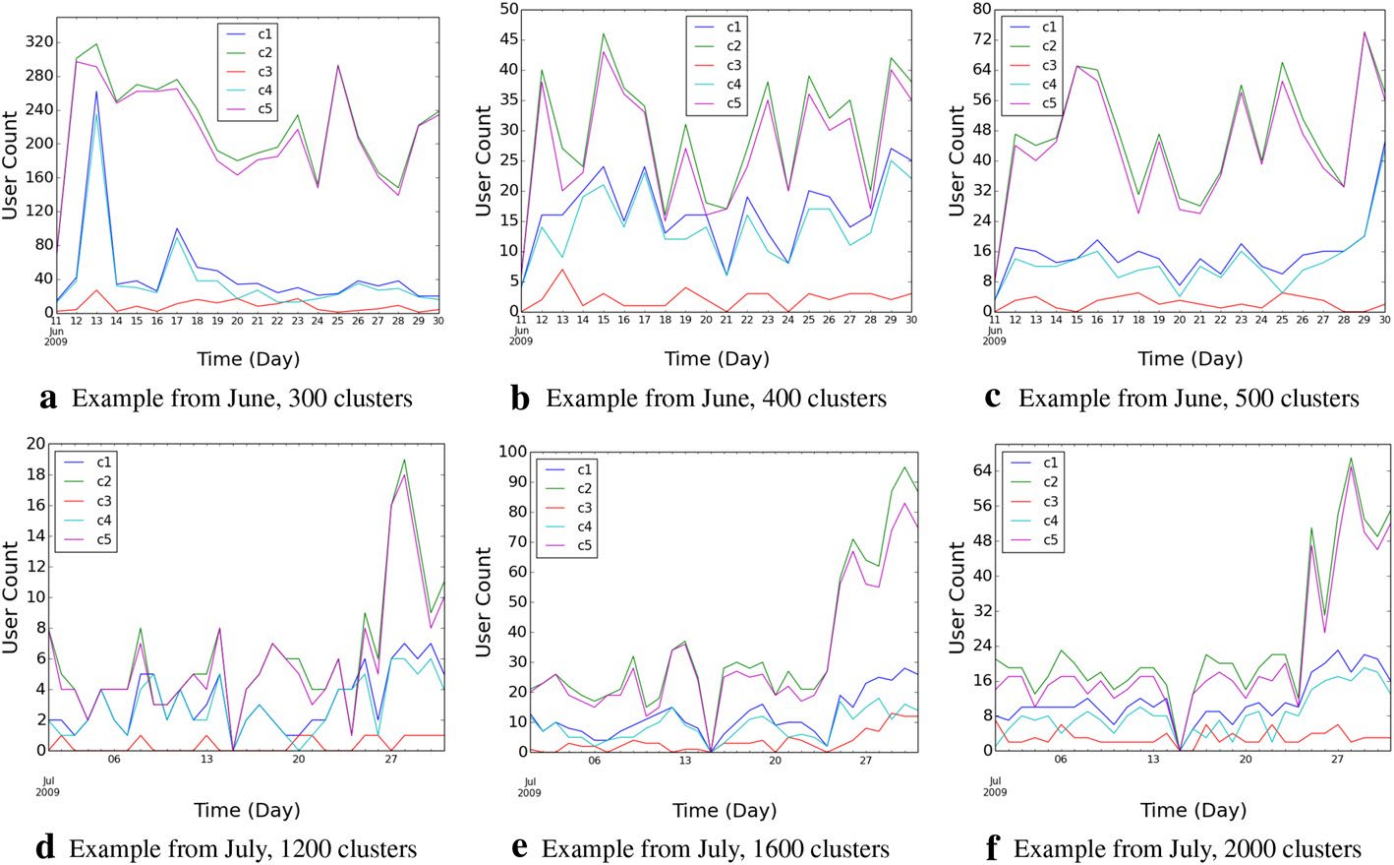
User distribution with respect to using dominant versus other hashtags. C1 is the count of users using dominant hashtags, C2 is the count of users using non-dominant hashtags, C3 is the count of users using both dominant and non-dominant hashtags, C4 $\leftarrow$ (C1–C3) is the count of users using dominant hashtags only (but do not use non-dominant hashtags at all), C5 $\leftarrow$ (C2–C3) is the count of users using non-dominant hashtags only (but do not use dominant hashtags at all)

Figure 4 shows the distribution of users using dominant versus non-dominant hashtags. As seen consistently, the number of users using dominant hashtags is consistently and significantly lower than those using non-dominant hashtags, over almost all the time slots, across all the data. And yet, by definition, dominant hashtags are the prominent ones, that govern the discussion topics. This shows that, a relatively smaller number of active participants in the topic, tend to be the “main voices” of that topic, and during this period, they tend to use the same hashtag. On the other hand, other users that also participate in a given topic tend to use a mixture of the dominant as well as non-dominant hashtags. This shows that *interested* users drive topic lifecycles.

**Fig. 5:**
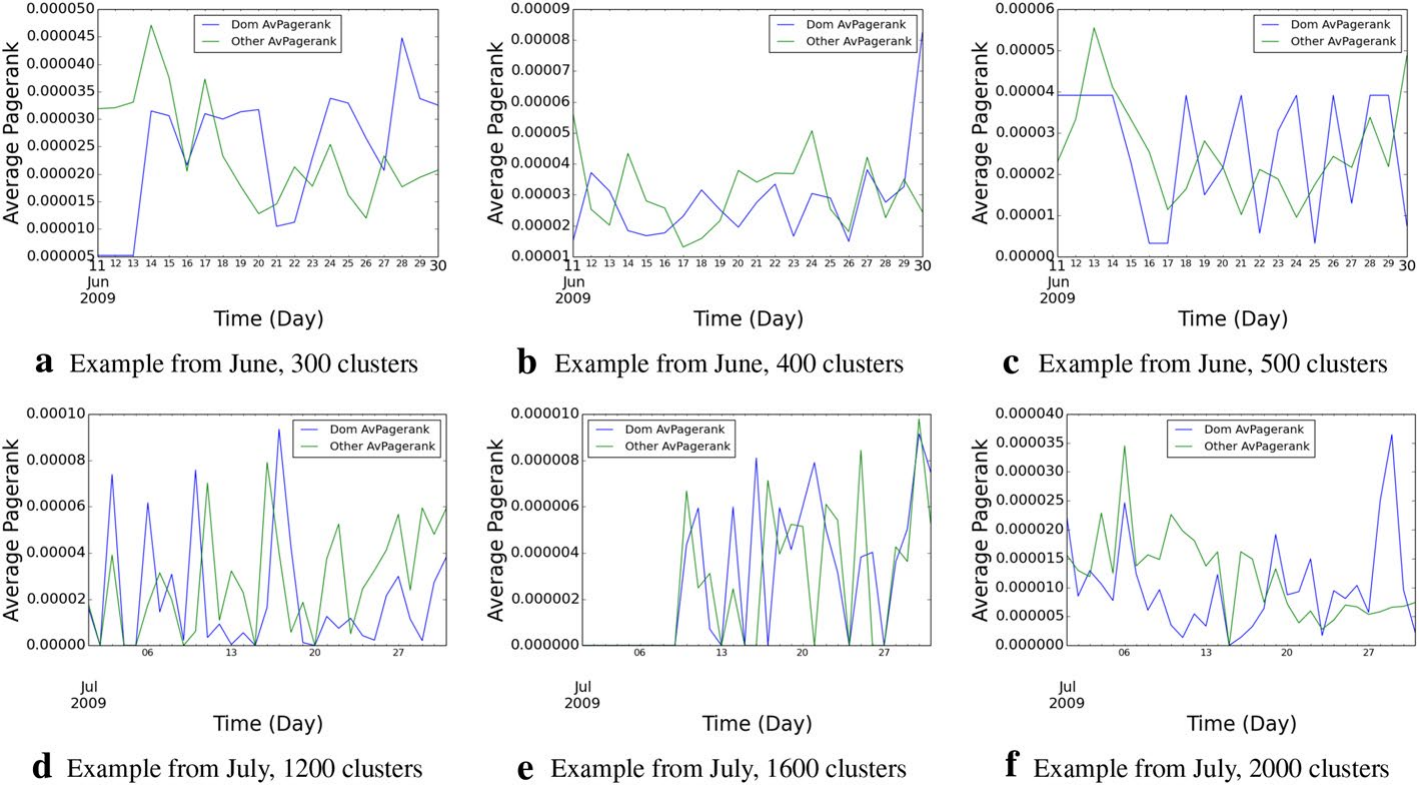
User influence computed using Page Rank. *Dom AvPagerank* denotes the average Page Rank of users using dominant hashtags at a given time slot, and *Other AvPagerank* denotes the average Page Rank of users using non-dominant hashtags at the same time slot

Figure 4 indicates that there is no clear trend in Page Rank pattern with respect to hashtag usage. While at some of the time slots, the average Page Rank of the users of the dominant hashtags is the highest, at other times, the reverse is true. The mix of the two also appears to be completely random. This shows that *influence* of users do not play a significant role in determining the course topic lifecycles on Twitter. Combining with the earlier observations, we make the following key inference: **topic lifecycles in Twitter are driven by user’s** *interests* **, and not by their** *influence* (Fig. 5).

**Fig. 6:**
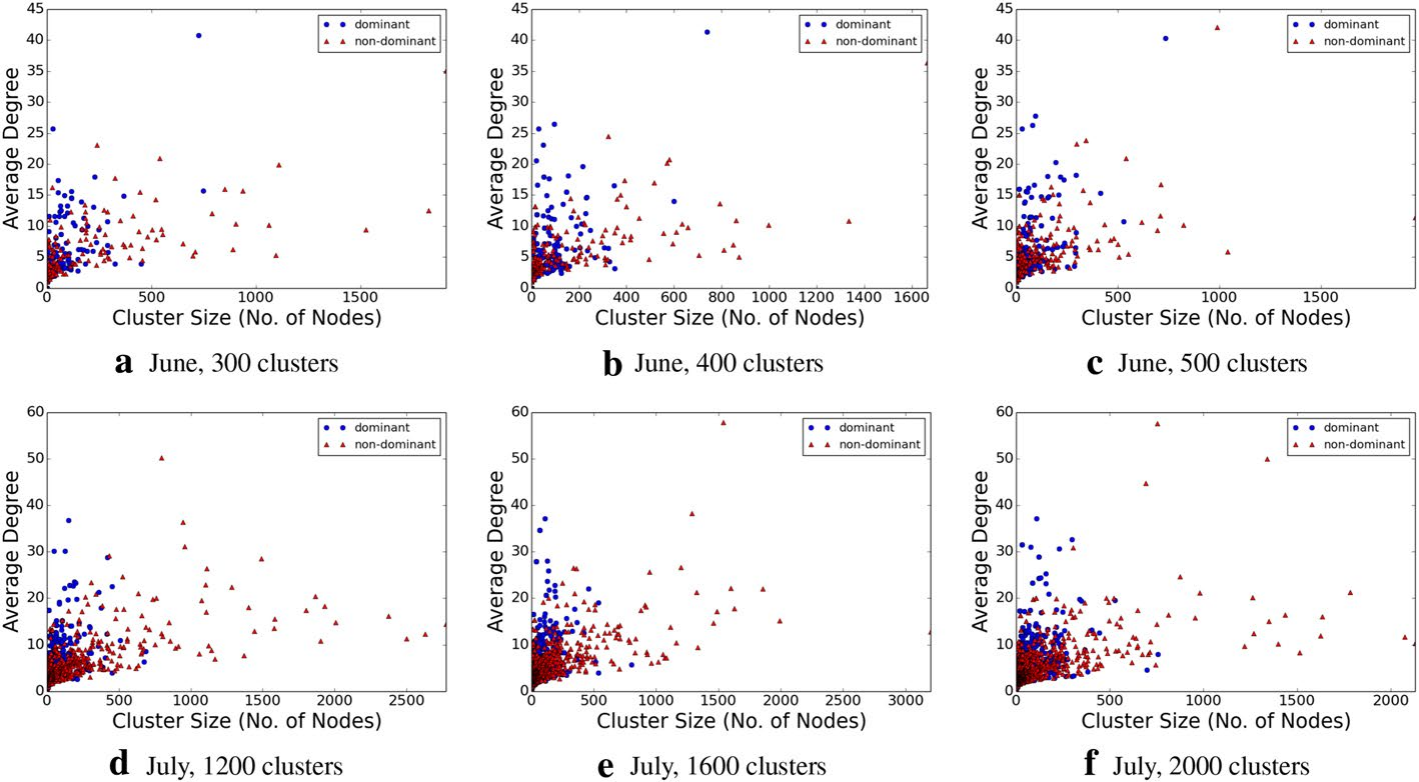
Average degree of the users using only dominant hashtags and of users using only other (non-dominant) hashtags

For each topic, we construct a pair of induced subgraphs. We construct one induced subgraph, by selecting all the users that have used only dominant hashtags in at least one time slot (and not used any non-dominant hashtag in that time slot), and retaining their social edges. Conversely, we construct another induced subgraph, by selecting all the users that have used only non-dominant hashtags in at least one time slot (and not used the dominant hashtag in that time slot), and retaining their social edges. We find the average degrees of the pairs of the induced subgraphs, and plot that against the number of participating nodes in the respective subgraphs, in Fig. 6. While due to the larger number of users of non-dominant users we find a large number of the non-dominant hashtags to have a large number of nodes, but the distribution of majority of the average degrees appear to be similar across the two different types of induced subgraphs. This, combined with our earlier findings, together indicate that, topic lifecycles remain active socially morphing from one topic to the other, but the driving users drive them from their similarity of interests.

In the following subsection, we explore the characteristics of the overall graph, based upon its social connection properties.

### Graph-level characteristics

**Fig. 7:**
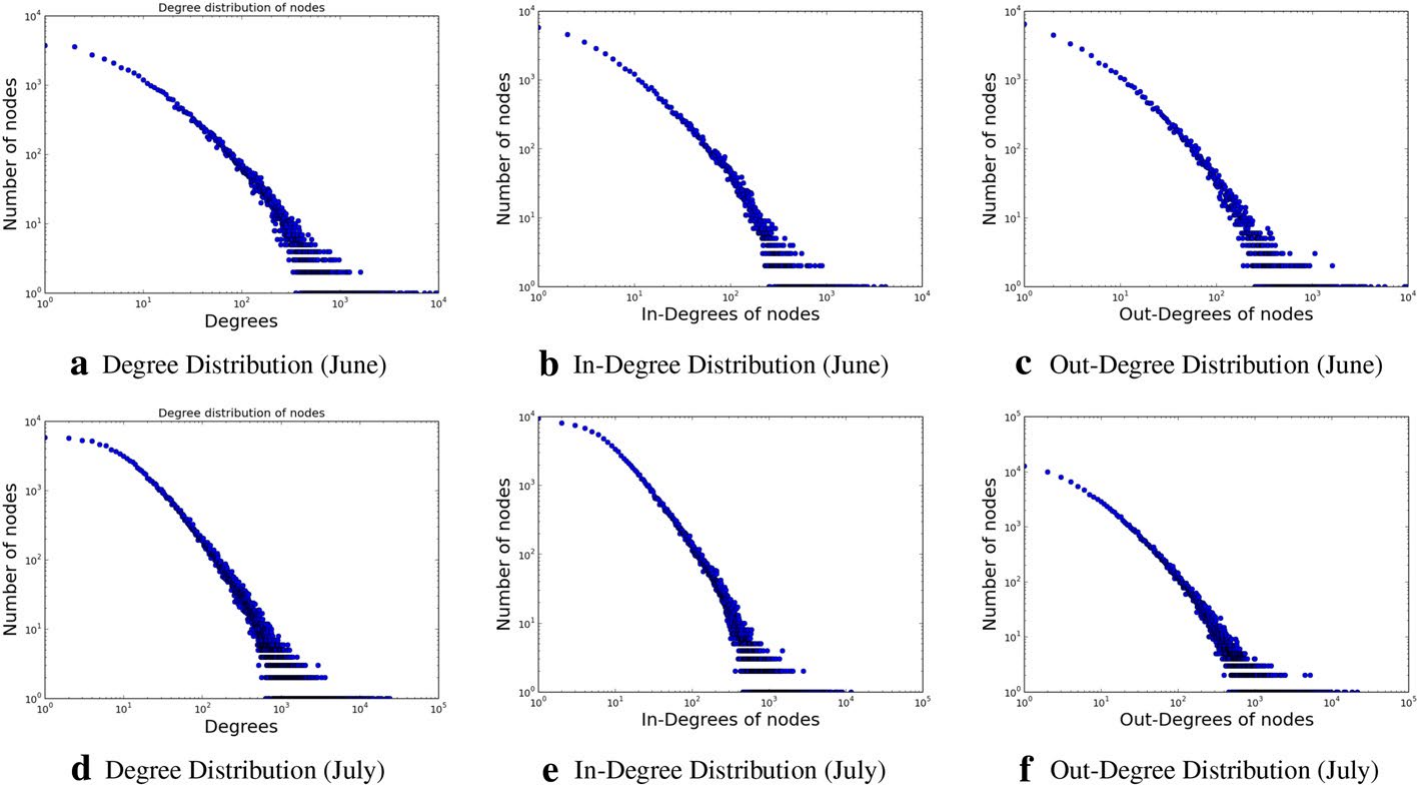
Overall, in-degree and out-degree distribution for the two data sets

We perform a graph-level characteristic study, constructing an induced subgraphs for each topic, from the original social connections graph. An edge is drawn in the induced subgraph if there is a social edge between a pair of users. Effectively, this gives rise to a topic lifecycle-specific network subgraph. Thus, the graph gets constructed. The input data are characterized by an exploration of the degree distributions, as shown in Fig. 7. We find the degree distributions to form a long tail, as observed by earlier studies too, e.g., Nanavati et al. [26].

**Fig. 8:**
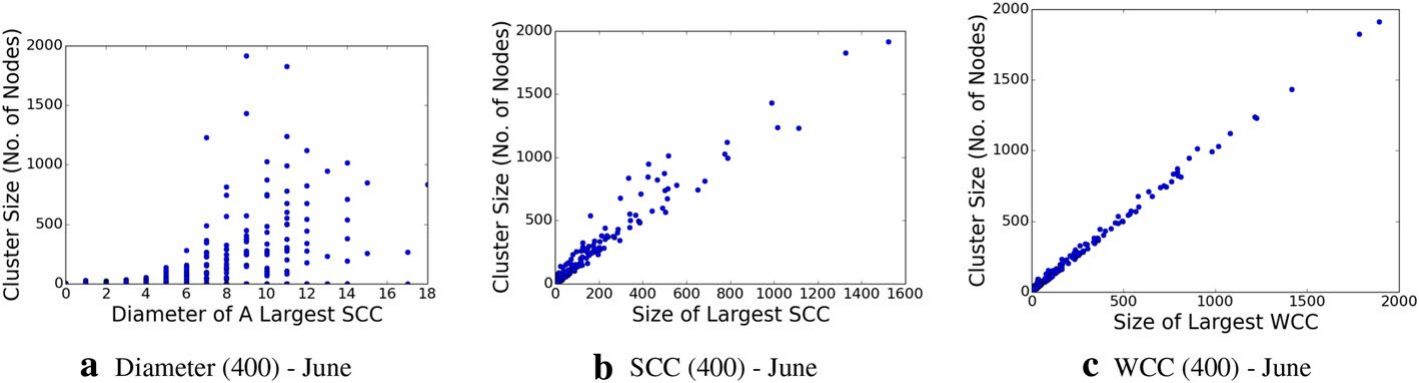
Network graph characteristic properties for the 400-topic granularity in June data

**Fig. 9:**
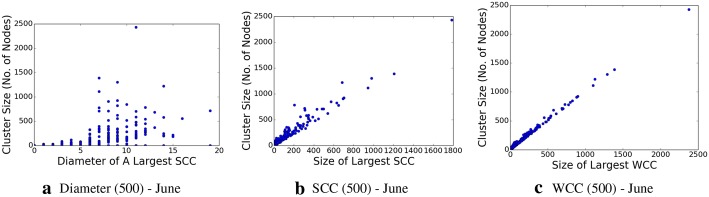
Network graph characteristic properties for the 500-topic granularity in June data

**Fig. 10:**
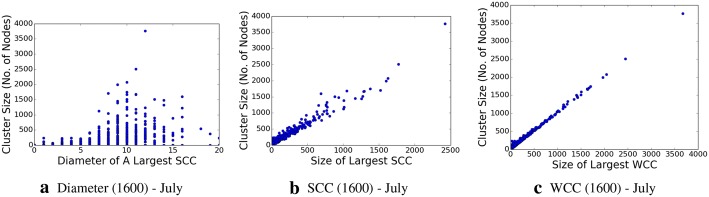
Network graph characteristic properties for the 1600-topic granularity in July data

**Fig. 11:**
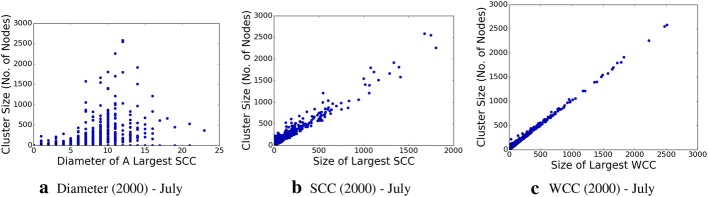
Network graph characteristic properties for the 2000-topic granularity in July data

We determine the graph diameter, as well as the connected components, both the strongly connected components and weakly connected ones, within this subgraph, which are shown in Figs. 8, 9, 10, and 11, respectively. The properties, and especially the presence of large strongly connected components (SCC) in addition to weakly connected components (WCC), as well as the relatively low diameters of the SCCs, indicate the presence of strong social connectivity in the graph.

Overall, combined with the observations and inferences made at all the earlier stages of this paper, we infer that, *topics morph over time using semantically related hashtags, over socially well-connected graphs, driven by user interests, but without much effect of user influence*.

Please note that, we use the Python “NetworkX” package to compute all the graph properties.

## Discussion

### Twitter topics: hashtag clustering versus other models

Multiple techniques exist in the literature for finding topics on Twitter. These include simple hashtag-based models [6], burst-of-keywords-based models [7], Latent Dirichlet allocation (LDA) [5]-based models, and hashtag semantic embedding distance and temporal gap-based clustering models [22]. Since the current work is the first to establish a baseline for lifecycle of topics and user role towards such lifecycle, we limit the scope of the current to one model of topic finding. Since the state-of-the-art in the literature has moved to word-embedding-based semantic-temporal topic detection, we have used this as the underlying technique for identifying topics, and have performed our study of topic lifecycle modeling as well as characterization of the role of participants using the topic clusters derived thereof. In the future, using our approach, we propose to conduct a separate full-fledged study, wherein we shall explore the impact of having different topic models (such as LDA, hashtag embedding, individual hashtags, bursty keywords, etc.) for analyzing topic lifecycles and investigating the roles of participants towards such evolutions.

### User influence, user interest, and topic lifecycle

Our work elicits a first-of-its-kind observation in the space of Twitter topic lifecycle analysis—the lifecycle and hashtag morphing are an effect of user interest, not influence. We observe the user influences (captured by Page Rank) to be a mixture of the dominant and other hashtags, where a dominant hashtag is defined as the most prominent hashtag of a topic at a given time slot. On the other hand, the dominant hashtags are always produced by a relatively much smaller number of users participating in the topic, while the rest of the users, although larger in number, tend to spread their usage over other hashtags. Dominant hashtags change with time as part of the evolution of the topic over hashtags; however, the characteristic of a topic (hashtag cluster) to be governed primarily by a small number of interested users, instead of a large number of users or users with high influence, remains unchanged. This is one of the key takeaways of the current paper.

Overall, we infer that *topics morph over time using semantically related hashtags, over socially well-connected graphs, driven by user interests, but without much effect of user influence*. This is a novel insight proposed by our work. In the future, it will be interesting to investigate whether any distinction exists between topics that are spontaneously born within social networks, versus topics that emerge out of influence (such as, preferential messages, advertisements, etc.).

## Conclusion

In this work, we modeled the lifecycle of topics on Twitter, using a model of topic detection that has not been used in this context earlier in the literature. The topic is determined as a cluster of hashtag, where the clustering is carried out using semantic similarity of containing tweets and temporal occurrence of those tweets. In the process, we observed how topics morph from one to the other over time, on multiple real-life data sets, over varying intensities of presence. We also observed how at different points of time, different hashtags dominate the hashtag (topic) clusters. In the process, we assessed the influence of the users using the different hashtags, for the dominating as well as the non-dominating hashtags for each cluster. We observed the power of the masses over individual influence in the social network settings across the data sets—the hashtags used by the majority tends to dominate the hashtag clusters, and the hashtags used by the influential users are seen to be non-dominant. Our work is the first of its kind, that analyzes the roles of users in shaping up the lifecycle of topics, and determining whether or not topics morph over time via hashtags or die down. We infer that topic lifecycles are governed by user interests, and not by user influence. The work can be used in social marketing and information spread modeling applications. In the future, we propose to use different measures of user influence as well as different semantic similarity and temporal thresholds, to refine the empirical understanding and obtain an improved solution.

## Footnotes

1 https://snap.stanford.edu/data/twitter7.html.

2 http://an.kaist.ac.kr/traces/WWW2010.html.
